# Endometriosis Gene Expression Heterogeneity and Biosignature: A Phylogenetic Analysis

**DOI:** 10.1155/2011/719059

**Published:** 2011-12-13

**Authors:** Mones Abu-Asab, Ming Zhang, Dennis Amini, Nihad Abu-Asab, Hakima Amri

**Affiliations:** ^1^Laboratory of Immunology, Section of Immunopathology, National Eye Institute, National Institutes of Health, Bethesda, MD 20892, USA; ^2^Department of Biochemistry and Cellular & Molecular Biology, Georgetown University Medical Center, Washington, DC 20007, USA; ^3^Department of Obstetrics and Gynecology, Georgetown University Hospital, Washington, DC 20007, USA; ^4^Armidale Rural Referral Hospital, University of New England and The University of Newcastle, Armidale, NSW 2351, Australia

## Abstract

Endometriosis is a multifactorial disease with poorly understood etiology, and reflecting an evolutionary nature where genetic alterations accumulate throughout pathogenesis. Our objective was to characterize the heterogeneous pathological process using parsimony *phylogenetics*. Gene expression microarray data of ovarian endometriosis obtained from NCBI database were polarized and coded into derived (abnormal) and ancestral (normal) states. Such alterations are referred to as *synapomorphies* in a phylogenetic sense (or biomarkers). Subsequent gene linkage was modeled by Genomatix BiblioSphere Pathway software. A list of clonally shared derived (abnormal) expressions revealed the pattern of heterogeneity among specimens. In addition, it has identified disruptions within the major regulatory pathways including those involved in cell proliferation, steroidogenesis, angiogenesis, cytoskeletal organization and integrity, and tumorigenesis, as well as cell adhesion and migration. Furthermore, the analysis supported the potential central involvement of ESR2 in the initiation of endometriosis. The pathogenesis mapping showed that eutopic and ectopic lesions have different molecular biosignatures.

## 1. Introduction

In the USA, 10–20% of women suffer from endometriosis, with 40% developing infertility. It is a public health issue with a patient's medical costs approximately 63% higher than those of the average woman [1]. The etiology and pathophysiology of endometriosis remains poorly understood. The hypothesis of retrograde menstruation is the oldest and most widely accepted. However, it fails to explain why some women develop endometriosis while others do not, given that some degree of retrograde menstrual flow is experienced by all women [2]. Thus, other factors such as immunology [3, 4], genetics [5], and the environment [6] may play a role in the mechanism of disease development.

The genetic theory dates back to the first formal genetic study published in 1980 by Simpson et al. [5]. Numerous findings since then support a *polygenic multifactorial inheritance* of endometriosis caused by an interaction between multiple genes with the environment. Several studies from the OXGENE (Oxford Endometriosis GENE) group confirmed an inheritance component to endometriosis. Specifically, in one report of 100 families with endometriosis from 6 different countries, 19 mother-daughter pairs and 56 sibling pairs shared the disease [7].

A diagnostic method that screened for genetic profiles or candidate genes may benefit the patient by detecting disease earlier, improving patient quality of life, discerning genetic predisposition, lowering costs, and reducing the need for invasive laparoscopic investigations.

Though not yet completely understood, numerous studies show a correlation between the occurrence of endometriosis and ovarian cancer [8–10]. Both diseases share pathogenic factors such as familial predisposition, genetic modifications, immunologic abnormalities, uncontrolled angiogenesis, and hormonal disturbances [11]. Malignant transformation of endometriosis has been reported [2, 12]. It is hypothesized that ectopic glands may expand monoclonally; however, this phenomenon is not yet clearly defined [13]. Elucidation of a cellular continuum from benign endometriosis to malignancy requires more research and a greater understanding of common mutational events.

Molecular processes involved in disease development share aspects of evolutionary transformation such as genetic mutations, clonal propagation, irreversible gene expression, and shared derived genetic alterations. Sarnat and Netsky first put forth the concept of disease etiology by evolutionary criteria in 1984 [14] whereby disease is viewed as an accumulation of genetic mutations. In this study, we sought to identify a genomic biosignature(s) for endometriosis using a newly developed evolution-based parsimony phylogenetics approach for gene expression microarrays data [15, 16] of endometriosis patients in order to stratify individual cases based on the molecular change, model the disease based on the level of patients' gene expression profiles, and identify affected molecular pathways involved in the disease process.

## 2. Methods

Gene expression microarray datasets of endometriosis patients, GSE7305, from NCBI's Gene Expression Omnibus database (http://www.ncbi.nlm.nih.gov/geo/) were used in the study [17]. Briefly, the datasets, submitted to NCBI by Hever and colleagues and successfully published in the Proc. Natl. Acad. Sci. USA [17], comprised 10 ovarian endometriosis and 10 matched eutopic endometria from the same patients using Affymetrix Human Genome U133 Plus 2.0 gene array. Polarity assessment was carried out using UNIPAL (Universal Parsing Algorithm) [18] by coding the expression values into ancestral (unchanged) and derived (deregulated/abnormal) states. Unchanged values were coded as zero (0) and altered/deregulated values as one (1), thus transforming the original expression values into a qualitative binary matrix of 0's and 1's [18]. Hierarchical classification through parsimony phylogenetic analysis was carried out with MIX, the maximum parsimony program of PHYLIP ver. 3.57c to produce cladograms [19]. TreeView was used to redraw the cladogram for final illustration [20].

Clonal alterations (or shared derived expression states, named synapomorphies in the field of phylogenetics or biomarkers in the biomedical field) were utilized to delimit a natural group of related specimens, termed a “clade.” The tree-like diagram, the cladogram, is the classification hypothesis that models the relatedness between the specimens. The full list of synapomorphies circumscribing the diseased specimens was extracted and analyzed without *a priori* selection. The analysis modeled the patterns of change occurring in the gene expression data set, classified specimens, and mapped the molecular events of altered pathways.

The synapomorphies were then modeled by Genomatix BiblioSphere Pathway Edition software version 7.2 for gene linkage. Genomatix BiblioSphere is data-mining software that extracts and analyzes gene relationships from literature databases (primarily NCBI PubMed) and genome-wide promoter analysis. The Genomatix collection of gene names and synonyms are supplied by NCBI Locust Link. We used this program to model our synapomorphies to reveal gene linkage and the affected pathways of pathogenesis. The gene maps have been filtered with respect to abstract cocitation level, B0.

## 3. Results

**Figure 1:**
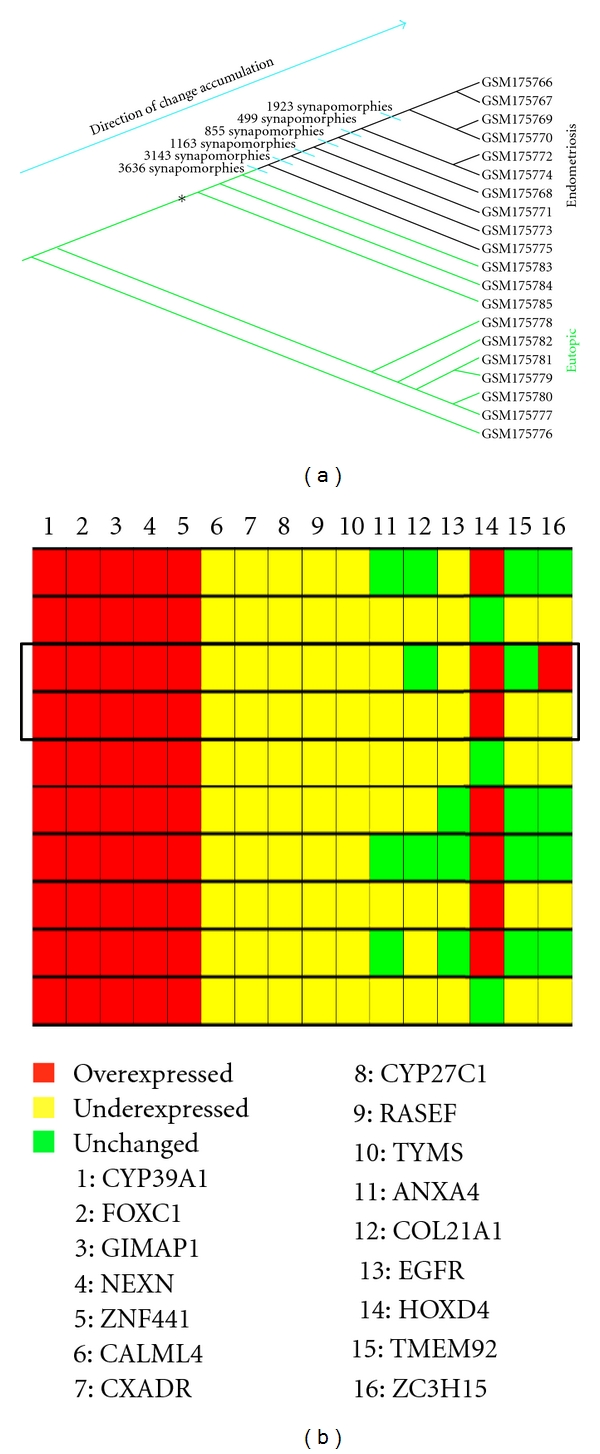
Cladogram and a corresponding heat map of selected genes. (a) A most parsimonious cladogram depicting a hierarchical classification of 10 ectopic endometriosis and 10 matched eutopic endometrial specimens from the same patients. The number of clonal gene expression aberrations is located at the crossing bar, and the directionality of change from eutopic endometrium to ectopic endometriosis specimen is indicated by an arrow. The transitional zone is denoted by the asterisk. (b) Heat map of 16 selected genes corresponds to the 10 endometriosis specimens and exemplifies heterogeneous gene expression profile of the endometriosis specimens. Synapomorphies are the aberrant clonal gene expressions that are shared by the specimens placed at the nodal point (at the bifurcations). The cladogram models the cumulative genetic change; it quantifies the severity of molecular disruption and points out the direction of change accumulation along the main axis of the cladogram in a hierarchical mode. The horizontal frame denotes the heterogeneity of the 16 genes highlighted within the heat map for two of the specimens. Note that the heat map is arranged to line up directly with the corresponding endometriosis specimens located on the adjacent cladogram.

The maximum parsimony phylogenetic program, MIX, produced one most parsimonious cladogram (Figure 1(a)). The cladogram has a directionality that reveals the amount of accumulated shared derived (abnormal) expressions from the base up (from eutopic endometrium to ectopic endometriosis specimens). The endometriosis specimens have the highest number of abnormal gene expressions. The analysis revealed that all the endometriosis specimens share 3,636 synapomorphies (or shared derived (abnormal) gene expressions) that distinguish them from the eutopic endometrium specimens. Synapomorphies are additive and accumulate for specimens positioned higher along the main axis of the cladogram. Thus, the additional 1,923 synapomorphies characterizing the highest clade consisting of 4 specimens (GSM175766, GSM175767, GSM175769, and GSM175770) share the greatest amount of deregulated genetic information that is most specific to these four subjects. Within the directionality or continuum of change, eutopic endometrium specimens (GSM175783–85) that may be susceptible to developing into endometriosis are located between the largest eutopic endometrium clade (GSM175777–78) and first endometriosis clade (GSM175775). The cladogram identified transitional patterns from eutopic endometrium (Figure 1(a)—lower, green) to endometriosis (Figure 1(a)—upper, black) specimens (GSM175783-85); these specimens separated from the majority of eutopic endometrium specimens and formed a transitional zone closer to the beginning of endometriosis specimens' clade. This analytical method can graphically delineate and molecularly represent the progression of endometriosis through the accumulation of changes in gene expression.

The modeling of gene expression heterogeneity is illustrated in the heat map in Figure 1(b). The heat map depicts overexpressed (red), underexpressed (yellow), and unchanged (green) gene expressions of 16 selected genes in all ten endometriosis specimens and their relative expression pattern per specimen, thus demonstrating the differential expression of genes across specimens.

The expression of ZC3H15, taken as an example (Figure 1(b), gene number 16), typifies the heterogeneity of single gene expression among the 10 endometriosis specimens. Thus, among the endometriosis patients, ZC3H15 has a heterogeneous expression: while it is unchanged in 4 specimens (GSM175766, GSM175774, GSM175768, and GSM175773), it is overexpressed in GSM175769 and underexpressed in 5 specimens (GSM175767, GSM175770, GSM175772, GSM175771, and GSM175775). Furthermore, the horizontal frame denotes the heterogeneity of several of the 16 genes highlighted in the heat map even within two specimens in close proximity on the cladogram that share a multitude of synapomorphies.

**Figure 2:**
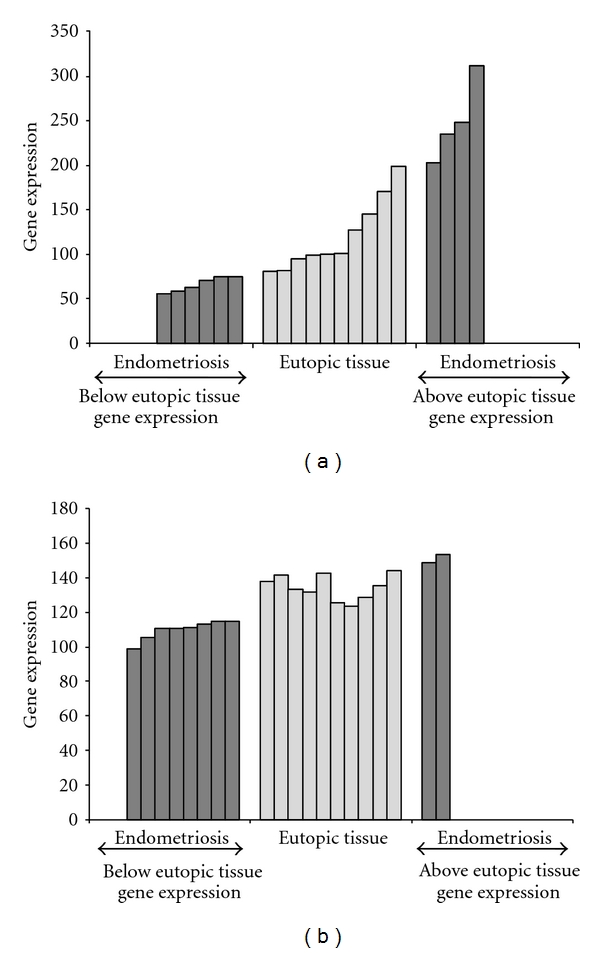
Parsimony phylogenetics identifies expression heterogeneity of single genes. (a) LCN2 (lipocalin 2) and (b) MYBBP1A (MYB binding protein (P160)) depict the dichotomous (under and over) gene expression as well as heterogeneity within the range of gene expression of the specimens.

The robustness of parsimony phylogenetics to model genetic heterogeneity is further illustrated in Figure 2. Lipocalin 2 (LCN2) and MYB binding protein (P160, MYBBP1A) are two examples of genes with dichotomous expression (above and below their gene expression range of normal specimens) as well as heterogeneity within the normal range of gene expression (Figure 2(a)). MYBBP1A appears to be tightly regulated as a slight deviation from the normal range which seems to induce a pathological state (Figure 2(b)).

 The functional and regulation relationships of the differentially expressed genes were assessed using Genomatix BiblioSphere (http://www.genomatix.com/). This analysis focused on the 1,923 synapomorphic genes of the highest clade (GSM175766, GSM175767, GSM175769, and GSM175770) to yield the greatest wealth of genomic insight into the pathology of endometriosis. Groups of shared derived genes were entered into Genomatix BiblioSphere including underexpressed and overexpressed. We analyzed the gene maps for prominent nodes as well as their central and extended linkages.

**Figure 3:**
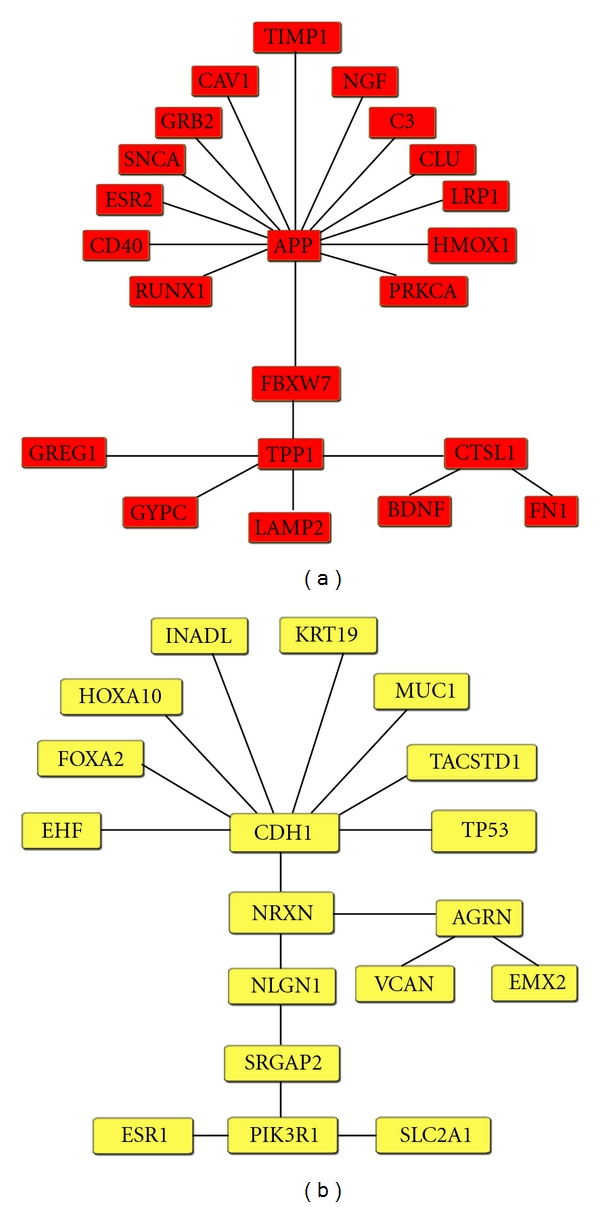
Gene linkage map for the over- (a) and underexpressed (b) genes filtered separately at the B0 level using Genomatix BiblioSphere. Gene list was obtained from the 4 specimens (GSM175766, GSM175767, GSM175769, and GSM175770) at the upper end of cladogram in Figure 1(a).

Out of the 1,923 gene synapomorphies aforementioned, 583 overexpressed, coded in red (Figure 3(a) and full gene listing in Supplemental Material (1) see Supplementary Materials available on line at doi: 10.1155/2011/719059) and 459 underexpressed genes, coded in yellow (Figure 3(b) and full gene listing in Supplemental Material (2)) were modeled.

**Figure 4:**
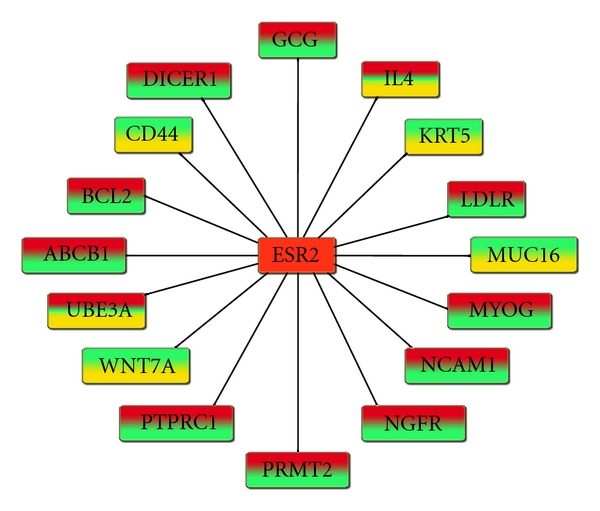
Gene linkage map of the genes at the node of the transitional zone (marked by an asterisk in Figure 1(a)) constructed by Genomatix BiblioSphere. The overexpressed genes coded in red, underexpressed in yellow, and unchanged in green were all combined and filtered at the B0 filter level. The color coding reflects the gene expression heterogeneity among specimens and the power of parsimony phylogenetics to reveal the dynamic disease process. The biosignature is centered around ESR2 as the potential major player influencing the gene network and change to endometriosis.

**Figure 5:**
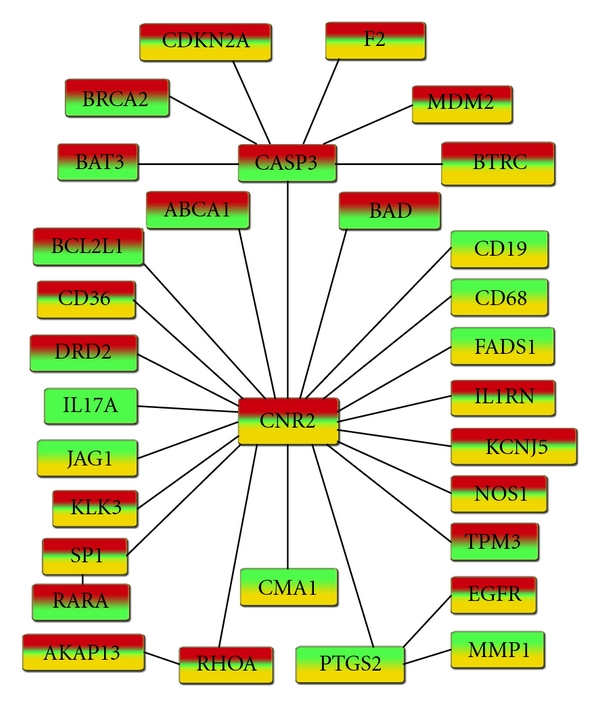
Gene linkage map of the genes at the lower clade (see Figure 1(a)) composed of seven endometrial eutopic specimens (GSM175776–82) constructed by Genomatix BiblioSphere set at the B0 filter level. The overexpressed genes coded in red, underexpressed in yellow, and unchanged in green were all combined and filtered at the B0 filter level. The color coding reflects the gene expression heterogeneity among specimens and the power of parsimony phylogenetics to reveal the dynamic disease process. The biosignature is centered on CNR2 and CASP3 as the potential major players influencing the gene network.

The cladogram in Figure 1 shows a group of three in-tandem specimens (GSM175783–85) that forms a transitional zone between eutopic endometrium and ectopic endometriosis. This clade was circumscribed by 707 synapomorphies. The pathway network analysis pointed out to the overexpressed ERS2 as the central deregulated gene affecting other gene nodes (Figure 4). This pathway analysis showed that the gene network was also different from the lower clade of the eutopic endometrial specimens (GSM175776, GSM175777, GSM175778, GSM175779, GSM175780, GSM175781, and GSM175782) (Figure 5).

## 4. Discussion

Parsimony phylogenetics, an evolution-based bioinformatic paradigm, revealed deregulated clonal expressions within ectopic endometriosis as compared to eutopic endometrium specimens. This analytical method achieved several goals: construction of the molecular disease boundaries and pathways' aberrations, stratification (subtyping) of disease, detection of early disease stages, suggestion of potential therapeutic targets, and identification of the biosignature (profile) of diseased specimens.

The comprehensive parsimony phylogenetics analysis revealed an extensive list of shared derived (deregulated/abnormal) expression states—or synapomorphies in a phylogenetic sense (biomarkers in a biomedical sense), which showed the extent of heterogeneity among specimens. Furthermore, it identified dichotomously expressed asynchronous genes (DEA) among endometriosis specimens [16]; these are gene expression values that are above and below the range of gene expression of the eutopic endometrium specimens (Figure 1(b)). Each DEA gene splits the specimens into two groups, thus showing the *heterogeneity* that exists among endometriosis specimens. This pattern was first reported by Lyons-Weiler et al. [21] and termed DEA by Abu-Asab et al. This phenomenon was designated *dichotomously expressed asynchronicity* to reflect its two-tailed distribution and deviation from the expression range of the outgroup [16]. While *F-* and *t-*statistics as well as fold change may not consider DEA genes significant or include them within the list of differentially expressed genes [21], the polarity assessment algorithm assesses each value as either *derived* or *ancestral,* thereby revealing the gene's status in relation to the gene profile of the outgroup [16].

LCN2 and MYBBP1A heterogeneous expression as DEA genes illustrates the complexity of this disease. LCN2 is known as a marker from benign to pre- and malignant ovarian tumors and may be involved in progression of epithelial ovarian malignancies. It is also an epithelial inducer in *Ras* malignancies and a suppressor of metastasis [22]. Upregulated in ovarian cancer cells, it may be involved in the progression of epithelial ovarian malignancies [23]. Our results showed that 4 specimens exhibited LNC2 overexpression which could explain the risk of progression of endometriosis from a benign to malignant condition in some patients [13, 24].

MYBBP1A is a novel NF-kappaB corepressor of transcription and DNA-directed polymerase activity [25]. Associations between the p160 coactivator proteins and endocrine resistance have been described, involving the MAP kinase effector proteins Ets [26]. This corepressor gene expression appears to be tightly regulated as a slight deviation from the normal range appears to induce a pathological state.

### 4.1. Overexpressed Genes

From the list of overexpressed genes, we selected to discuss only a few among those reported in the literature as relevant to the pathogenesis of endometriosis.

The endocrine-gland-derived vascular endothelial growth factor (PROK1) has been shown to possess a paracrine role for prokineticins and their receptors in endometrial vascular function [27]. Endometriotic implants require neovascularization to proliferate and invade into ectopic sites, and such angiogenic factors are currently being targeted for novel medical therapeutics [28].

Caveolin-1 (CAV1) has been shown to negatively regulate the Jak-2 tyrosine kinase in mice [29] and the latter modulating the processes of cell proliferation, differentiation, and apoptosis [30].

Nerve growth factor (NGF) levels are higher in the follicular fluid of women with endometriosis [31]. Histological analysis of human deep innervating endometriosis (DIE) tissue showed strong expression of NGF in endometriotic glands and stroma of DIE which may play a role in the pathways involved in the intense pelvic pain that patients experience [32].

Hydroxysteroid (17-beta) dehydrogenase 11 (HSD17*β*-11) converts 5 alpha-androstane-3 alpha, 17 beta-diol to androsterone [33]. Expression analysis has revealed significant upregulation of enzymes involved in estradiol synthesis (i.e., aromatase, sulfatase, and all reductive 17 beta-HSDs), which indicates increased local levels of mitogenic estradiol and decreased levels of protective progesterone in endometriosis [34].

### 4.2. Underexpressed Genes

BCL-2-related ovarian killer (BOK) is a proapoptotic protein identified in the ovary [35] and functions as an essential mediator of p53-dependent apoptosis [36].

It is well established that the matrix metalloproteinase system (MMPs) plays an important role in the normal development of the endometrium. MMPs have also been implicated in the adhesive, invasive, and metastatic processes involved in endometriosis [37]. Both ectopic and eutopic endometrial tissues show altered levels of MMP and TIMP expression, favoring tissue invasion and remodeling.

Tumor protein 53 (TP53) regulates the cell cycle functions as a tumor suppressor and while its role in endometriosis remains unclear, there is evidence to support its apoptotic resistance and enhanced survival of endometrial cells in endometriosis [38]. TP53 was found to be overexpressed in epithelial cells in a considerable number of endometriotic lesions [39]. However, it was found that TP53 was insignificantly upregulated in endometriosis tissue when compared with control endometrium [40].

Estrogen plays a significant role in the maintenance and chronic bleeding of endometriosis. Estrogen receptor 1 (ESR1 or ERalpha) is the dominant receptor in the adult uterus and the major mediator of estrogenic effects. It plays a role in the hormonal deregulation and inflammation seen in this disease [41]. Steroid hormone receptors such as ESR are altered in endometriosis [42, 43].

The analysis of the combined pathway of over- and underexpressed genes, as summarized in Table 1, revealed that tissue inhibitor of metallopeptidase 1 (TIMP1) may participate in the process of invasion and tissue remodeling that is hypothesized to occur in the pathogenesis of endometriosis [44]. In endometrial carcinomas, Ephrin-B2 (EFNB2) expression may reflect or induce increased potential for growth and tumorigenicity [45].

Brain-derived neurotrophic factor (BDNF) levels are low in the follicular fluid of women with endometriosis and suggest that neurotrophins may contribute to the pathogenesis via aberrant oxidative stress mechanisms [31]. Shaco-Levy et al. (2008) found that levels of CDH1, MMP-2, and MMP-9 expressions were significantly higher in endometriosis as compared to endometrioid carcinoma, indicative of altered cell proliferation, migration, differentiation, angiogenesis, apoptosis, and host defense [46].

Increased levels of fibronectin 1 (FN1) by peritoneal macrophages in patients with endometriosis may contribute to the adhesion formation and associated reactive fibrosis seen in this disease and may influence the implantation of endometrial cells and their subsequent growth in the pelvis [47].

Phosphoinositide-3-kinase and RAS/RAF/MAPK pathways have been suggested to be involved in the initial development of endometriosis [48]. Intercellular adhesion molecule 1 (ICAM1) may play a role in the early implantation of peritoneal endometriosis [49].

### 4.3. Transitional Zone

While the analysis of the upper clade of the endometriotic specimens showed a particular biosignature, the analysis of the lower clade, composed of eutopic endometrial tissue of patients with endometriosis, revealed two distinct biosignatures, one specific to the lower clade and the other to the transitional zone. Although different sets of genes were identified, they are also involved in the control of inflammation, the immune response, apoptosis, cell proliferation, and lipid metabolism (Table 2). The chymase 1 gene (CMA1) found in mast cells has been shown to influence the inflammatory response by converting interleukin-1 beta into the active form, interleukin 1 [50]. The prostaglandin-endoperoxide synthase 2 gene (PTGS2/COX2) has been reported to play an important role in the inflammatory response through the production of prostaglandins [51]. Meanwhile, the cannabinoid receptor 2 (CNR2) has been shown to play an anti-inflammatory and antioxidative role in mice that have undergone chemotherapy [52].

Other studies have shown that member A of the Ras homolog gene (RHOA) can influence cell apoptosis in heart muscle cells [53]. The caspase 3 gene has also been found to induce apoptosis in cells when overexpressed [54], but possess a negative feedback mechanism as well to prevent excessive and potentially harmful mass cell death [55]. The other apoptosis-related gene is BAD; it could induce apoptosis through cleavage by caspases or inhibit apoptosis if the gene is overexpressed [56]. Finally, studies have found that the ABCA1 gene plays a major role in cholesterol transport across cell membranes [57]. This can greatly affect the synthesis of steroid hormones such as estrogen, which is well known to possess a strong stimulating effect on endometriotic growth [58]. Among the gene synapomorphies (biomarkers) identified is DRD2, which has recently been linked to eutopic and ectopic endometriotic lesions and suggested as a target to develop therapeutics [59].

### 4.4. Applications in Diagnosis and Prognosis

**Figure 6:**
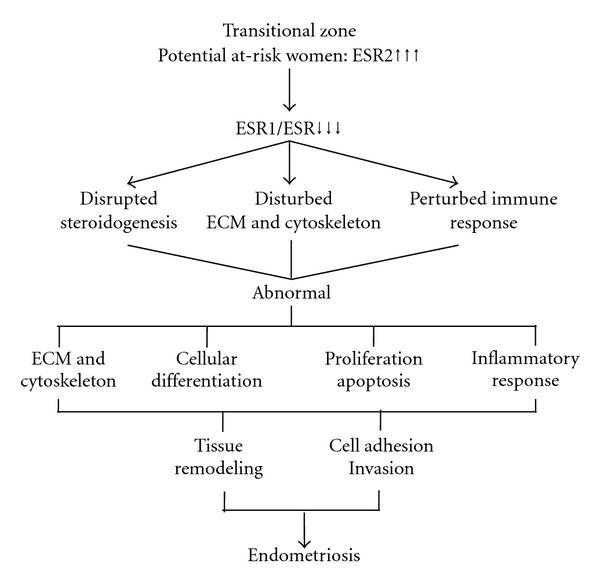
Diagram summarizing the central role of ESR2 in triggering the molecular cascade of cell and tissue dysfunction in the transitional zone, which could lead to endometriosis.

Expression profile of specimens at the border between eutopic endometrium and endometriosis specimens, interestingly, revealed that the overexpressed estrogen receptor 2 (ESR2) is a central linkage to other gene nodes. The transitional status of these specimens is highlighted by the mostly dichotomously expressed synapomorphies (Figure 4). This is an important finding because it shows that the overexpression of ESR2 could be the triggering step that initiates the deregulation of other key genes associated with inflammation, cellular matrix, immune response, growth factors, apoptosis, and others, thus leading to endometriosis (Supplementary Material 3). Indeed, several studies have reported high expression of ESR2 but lower levels of ESR1 in endometriotic tissue which caused a decrease in ESR1/ESR2 ratio [41, 60, 61] and which is in agreement with our findings (see also ESR1 in Figure 3). While Bulun and colleagues recently proposed a hypothetical model where the strikingly low ratio of ESR1/ESR2 could shift the stimulatory effect of estradiol on the progesterone receptor expression [62, 63], our study showed that the overexpression of ESR2 could precede the pathological and clinical signs of endometriosis; these potentially at-risk specimens grouped together closer to diseased specimens. The overexpression of ESR2 could be triggered by several factors ranging from genetic predisposition [64] to environmental exposures [65–67]. ESR2 polymorphism has been reported to play a role in endometriosis in various populations such as Brazilian [68] and Japanese women [69]. The disruption of ESR2 and the ensuing decrease of the ESR1/ESR2 ratio could be the culprit for the cascade of molecular events that initiates cellular deregulation and tissue remodeling associated with endometriosis (Figure 6). The screening for increased ESR2 expression could offer a diagnostic tool to identify women at risk of developing endometriosis.

It should also be noted that the endometrial tissue of women with endometriosis is different from the endometrial tissue of healthy women without the disease. For example, differences in proliferation of endometrial epithelial, stromal, and endothelial cells [70, 71], spontaneous apoptosis [72, 73], expression of cell adhesion molecules [74], and production of steroids and cytokines [74, 75] have been found. The limitation of our study is that we are restricted by the original design of published studies as deposited in the public domain. Although this dataset was limited to only 20 specimens, Hever and colleagues successfully published their findings [17] and made them available. It is important to note that endometriosis omics data available in the public domain is limited.

In summary, through this study, we have shown that the biosignature of the endometriotic lesion is different from that of the endometrial eutopic tissue. Furthermore, we have revealed a particular biosignature for specimens that are in a transitional state to develop uterine endometriosis. This study contributed a novel phylogenetic approach to modeling the molecular heterogeneity of endometriosis patients into a tree-like hierarchical cladogram that reveals the simultaneously deregulated gene expressions—also termed clonal or driver aberrations. This data-based analysis shows not only directionality of change from eutopic endometrium to ectopic endometriosis, but also its usefulness in categorizing specimens according to the accumulation of molecular changes, which can be applied in diagnosing or for screening patients at-risk for developing endometriosis. In addition to supporting the ESR1 to ESR2 ratio hypothesis on the initiation of endometriosis, we have shed light on new genes and pathways that were not previously described as significant to the pathology of endometriosis. This work is a necessary first step in examining novel gene networks by a biologically compatible method that could shed light on principal drivers of the disease development process.

##  Glossary of Phylogenetics Terminology Used in This Paper

Since the field of phylogenetics, already extensively used in biology, zoology, botany, virology, and parasitology for over 50 years, is new to the biomedical field, we think that providing a glossary would be useful to the reviewers and readers.

**Clade**

A group of specimens sharing one or more synapomorphies.

**Cladogram**

A graphic representation of relationships among specimens based on the synapomorphies (shared derived characters). The cladogram is a summary of trends that occur in the data while the upper part of it represents the specimens with highest amount of synapomorphies (shared mutations).

**Dynamic Classification**

A classification that has the capacity to incorporate new novel specimens without major alterations to its main groups.

**Outgroup**

The group of specimens used to polarize the ingroup values of gene expression into ancestral (plesiomorphic) and derived (apomorphic).

**Ingroup**

The group of specimens under study, for example, cancer specimens or endometriosis specimen in this study.

**Parsimony**

Means simplicity, the preferred hypothesis is the one requiring the least number of explanations (Occam's Razor). In the context of our work, the preferred phylogenetic tree is the tree that requires the least number of steps to construct it from the polarized data matrix.

**Polarity Assessment**

Also known as outgroup comparison. It is the basis of sorting out the data values (whether proteomic (m/z), or microarray expression values) into ancestral and derived. By using our algorithms (UNIPAL/E-UNIPAL), we transform absolute numbers from data values into polarized binary numbers (0/1), where zero (0) signifies ancestral and one (1) signifies derived.

**Predictive Classification**

A classification that reveals the characteristics (or profile/pattern) of a specimen when its place in the classification is known.

**Phylogenetic Classification [Phylogenetic Systematics]**

A classification that uses synapomorphies to delimit clades (i.e., monophyletic groups).

**Synapomorphy**

A shared derived protein or gene expression value in comparison with a number of normal specimens (the outgroup). A protein synapomorphy may have one of the following conditions: (1) a new novel protein, (2) a disappeared protein, (3) up regulated protein, (4) down regulated protein, and (5) asynchronously regulated protein (the m/z values are above and below the normals' range but not within the normals' range). A gene synapomorphy may have one of the following conditions: (1) overexpressed value above normals' range, (2) underexpressed value below the normals' range, (3) dichotomously asynchronous values, and (4) unmeasurable expression value.

## Supplementary Material

The supplemental material represents the listing of the 1,923 differentially expressed genes or synapomorphies characterizing the biosignature of the four endometriotic specimens of the upper clade in figure 1.

## Figures and Tables

**Table 1:** Gene list of over- and underexpressed genes summarized by function. These dichotomously expressed genes reflect the gene expression heterogeneity among specimens.

Function	Gene symbol				
Cell cycle	CAV1	CCNA2	DDR2	E2F2	GPC3
	GPC6	GTSE1	IFI16	MAD2L2	MAP2K6
	NCAPH	PTPN21	PTPN3	RBBP8	RPS6KA3
	SLBP	TGFBR3	TP53	TRMU	

Cell adhesion and migration	CDH1	CDH3	CLDN3	CLDN4	CLEC10A
	HOXA10	HOXA11	HOXB2	HOXB3	HOXB4
	HOXB5	HOXB6	HOXB7	HOXB8	HOXC4
	HOXC5	HOXC6	IQGAP1	LGALS8	TGFBR3

Apoptosis	IHPK2	BIRC5	BNIP3L	BOK	FAIM3
	IL24	MAP2K6	MAPK10	PAK6	RBMS3
	TP53	TPD52L1	WDR26		

Infertility/fertility	LEFTY2	PAK6	SPA17	SPAG1	

Immunity	BOC	BST2	CD40	CEACAM1	CLEC10A
	ICAM1	IGSF11	JAK3	NR3C1	PVRL3
	RIPK2	SIGLEC1	SIGLEC11	TNFSF13B	TSC22D3

Cell structure	ACTA2	ACTG2	ARHGAP25	ARL6IP5	CLDN5
	CNKSR1	COL10A1	COL3A1	COL4A3	COL8A1
	DCLK1	EMCN	ESR1	HS6ST2	IQGAP1
	ITGA11	ITGB8	KRT19	LAMA5	LAMB2
	LAMC2	LAMC3	LTBP2	MMP26	NID2
	PAK6	PCOLCE	PPFIBP1	SGCE	SIRPA
	SPC25	SPTBN1	TGFBR3	TNS1	VAPA

Iron	FTL				

Angiogenesis/invasion	ADAMTS3	ANGPT1	ANGPTL1	C9ORF47	ITGA7
	NRP1	NRP2	PROK1	S1PR3	TIMP1
	TIMP4				

Proliferation	ADAMTS18	CLDN11	CREG1	DOK5	DUSP4
	EHF	GPC6	IFI16	MAP3K1	MAPRE2
	NTRK2	PTPRB	TRAF4		

Steroid hormone regulation	AKR1C1	AKR1C2	CPE	CRYM	CYP11A1
	CYP2J2	CYP39A1	DIO2	ESR1	FST
	HSD11B1	NR3C1	PLTP	PTGER3	PTGFR
	PTGIS	RORA	STAR	VIPR2	

Tumor suppressor	DIRAS3	E2F2	FABP3	LYVE1	SMARCB1

Carcinogenesis	DOCK4	ERBB3	ESR1	FN1	HLA-C
	JAZF1	IGK	IGKC	NBR1	RECK
	TBX2				

Stress response	BDNF				

**Table 2:** Gene list summarizing the biosignature of the eutopic clade.

Function	Gene symbol				
Cell cycle	CDKN2A	PTGS2	EGFR	MDM2	F2
	BRCA2	BTRC			

Clotting/vascular integrity	F2	NOS1	RHOA	MMP1	SP1

Cell adhesion and migration	CD36	RHOA	MMP1	EGFR	CDKN2A
	F2	JAG1	PTGS2		

Apoptosis	MDM2	BAT3	BCL2L1	IL17A	RARA
	CASP3	BAD	AKAP13	PTGS2	EGFR
	CDKN2A	F2	RHOA		

Infertility/fertility	MMP1	EGFR	BRCA2	BCL2L1	PTGS2

Immunity	CD19	CD68	IL17A	IL1RN	RARA
	CMA1	CDKN2A	BAD	CD36	CNR2
	MMP1				

Inflammation	IL1RN	IL17A	CMA1	PTGS2	F2
	CNR2				

Cell structure	KCNJ5	TPM3	RHOA	EGFR	F2
	MMP1				

Carcinogenesis	TPM3	KLK3	EGFR		

Angiogenesis/invasion	JAG1	KLK3	PTGS2		

Cell proliferation	EGFR	CDKN2A	F2	BRCA2	MDM2
	BTRC	BAD	BCL2L1	JAG1	

Organogenesis	SP1	TPM3	MMP1	EGFR	BCL2L1
	DRD2	JAG1			

Steroid hormone regulation	RARA	RHOA			

Tumor suppressor	MDM2	BAT3	BRCA2	NOS1	

Stress response	FADS1	NOS1	RHOA		

Protein ubiquitination	BTRC	MDM2			

Lipid metabolism	ABCA1	FADS1	PTGS2	CD36	

Dopamine receptor	DRD2				

Ion transport	KCNJ5	SP1	RARA	DRD2	PTGS2

Glucose homeostasis	BAD	RHOA			

Cell debris removal	CD68	CD36			

Water and ion balance	DRD2				

Cell differentiation	FADS1	JAG1	SP1	RHOA	

## Abbreviations

UNIPAL: Universal Parsing Algorithm
DEA: Dichotomously Expressed Asynchronous genes.
