# Supplementary material for: Endometriosis Gene Expression Heterogeneity and Biosignature: A Phylogenetic Analysis
Source: Obstet Gynecol Int. 2011 Dec 13;2011:719059. doi: 10.1155/2011/719059 (PMC3238413; doi:10.1155/2011/719059)
Supplement: Supplementary file 1 — The supplemental material represents the listing of the 1,923 differentially expressed genes or synapomorphies characterizing the biosignature of the four endometriotic specimens of the upper clade in figure 1. [file 719059.f1.doc]

Supplemental 1: Overexpressed genes among the 1923 synapomorphies of the upper 4 specimens of endometriosis.

ABCA6

ABCA9

ACACB

ACO2

ACTA2

ACTG2

ADAMTS18

ADAR

ADCY9

ADD1

ADPRH

AEBP1

AGTR1

AHCTF1

AKAP12

AKR1B1

AKR1C1

AKR1C2

ALAD

AMHR2

ANGPT1

ANGPTL1

ANKRD57

ANKRD6

ANXA6

AOC3

AOX1

AP3S1

APOA1

APP

AQP1

AQP11

ARHGAP22

ARHGAP24

ARHGAP25

ARHGEF4

ARHGEF6

ARL6IP5

ARTN

ASH1L

ASTN1

ATP2A3

BAMBI

BBX

BDNF

BEX1

BEX4

BGN

BICD2

BNC2

BNIP3L

BOC

BRUNOL4

BST2

C10orf54

C14orf4

C16orf53

C17orf48

C1QTNF1

C1S

C21orf7

C3

C3AR1

C9orf5

CALD1

CAMK1D

CAMK1G

CAPN2

CASK

CASP1

CAV1

CAV2

CCDC80

CCL14

CCL26

CCPG1

CD163

CD22

CD302

CD40

CD47

CDGAP

CDH15

CDIPT

CENTA2

CFH

CFHR1

CHGB

CLDN11

CLDN5

CLEC10A

CLIP1

CLIP3

CLU

CMIP

CMYA5

COL10A1

COL4A3

COL6A2

COL8A1

COL8A2

COMMD6

CPE

CPEB1

CPNE8

CPZ

CREBL2

CREG1

CRIM1

CSTA

CTSL1

CUGBP2

CUL4B

CYLD

CYP11A1

CYP2U1

CYP39A1

D4S234E

DCLK1

DDEF1

DDEFL1

DDR2

DDX11

DHRS2

DIRAS3

DLC1

DLK1

DMD

DMN

DNAJB4

DNAJC21

DOCK4

DOK5

DPYD

DPYSL3

DSE

DTNA

ECM2

EDIL3

EEA1

EFEMP1

EIF4EBP2

ELK4

ELMO1

EMCN

EMILIN1

EMP2

ENAH

ENG

ENOX1

ENOX2

EPHA4

EPHX1

ESCO1

ESD

ESF1

ESR2

F8

FABP3

FABP4

FAM129A

FAM162A

FAM19A2

FAM46A

FAM49A

FAM62B

FBXW7

FCGR2A

FCGR2C

FCHSD2

FGF7

FGFR1

FGFR1OP2

FGL2

FHL2

FHL5

FIBIN

FLJ25076

FLRT3

FLT1

FMNL3

FMO1

FMO4

FMOD

FN1

FNDC4

FOXC1

FOXN3

FRZB

FST

FTL

FXYD6

FYCO1

FZD4

FZD7

GAPVD1

GAS1

GATA4

GATA6

GATM

GHR

GIMAP4

GJA5

GLIS2

GLS

GNA12

GNAQ

GNAZ

GNG12

GNG2

GPC3

GPC6

GPIHBP1

GPR1

GPR116

GPR126

GPR155

GRB2

GRIK5

GRK5

GYG1

GYPC

HCFC2

hCG_1998957

HEBP1

HEG1

HERC5

HMOX1

HOXC4

HOXC6

HOXD8

HS6ST2

HSD11B1

HSD17B11

HTRA1

ICAM1

IDS

IFI16

IFI44

IGHM

IGJ

IGLV@

IGSF11

IL13RA1

IL17RD

IL24

IL4R

ING2

INMT

INSIG2

IPO8

IQGAP1

ISCU

ITGA7

ITIH3

ITLN1

ITM2A

ITPR1

JAK3

JAM3

KCNQ4

KCTD12

KLF2

KLHL4

KPNB1

LAMP2

LDB3

LEFTY2

LGALS8

LHFP

LHX9

LIFR

LMNA

LMNB2

LNX1

LPAR4

LPIN1

LPIN2

LPP

LRP1

LRP4

LRRC2

LRRK2

LRRN3

LTA4H

LTBP3

LXN

LY96

LYVE1

MAG

MAML2

MAN1C1

MAP1A

MAPK10

MAPRE2

MATN2

MCAM

MCFD2

MDFIC

MEF2C

MEN1

MEOX2

MGCR

MKL1

MMRN2

MRAS

MRC1

MS4A1

MS4A4A

MS4A6A

MSRB3

MTCH1

MTM1

MTSS1

MYCBP2

MYH11

MYL9

MYLK

NAALADL1

NBEA

NBR1

NCAM1

NDE1

NEFH

NEGR1

NELL2

NFASC

NFIB

NFIX

NGF

NID2

NOD1

NOPE

NPR1

NR0B1

NR1H4

NR3C1

NRK

NTRK2

NUAK1

OBSL1

ODZ4

OMD

OPTN

OSAP

PALM2-AKAP2

PAPSS2

PBX3

PCSK7

PDE1A

PDE2A

PDE7B

PDGFD

PDGFRL

PDK4

PDLIM3

PDLIM5

PDZRN4

PEG10

PEG3

PEX19

PGM1

PHACTR3

PHF15

PHF17

PHLDB2

PICALM

PITX3

PLEC1

PLEKHA4

PLEKHA6

PLN

PLOD2

PLS3

PLSCR1

PLSCR4

PLTP

PLXNA2

PLXNA4

PMP22

PNOC

PODN

PON3

PPFIBP1

PPP1R12B

PPP1R1A

PPP1R3C

PPP2CB

PPP3CA

PRAGMIN

PRELP

PRKAR1B

PRKAR2B

PRKCA

PRKCSH

PROK1

PROS1

PROX1

PRSS35

PSD3

PTGER3

PTGFR

PTGIS

PTPN2

PTPN21

PTPRB

PTRF

PVRL3

QKI

QSOX1

RAB11FIP2

RAB12

RAB31

RAB3B

RAB7A

RAB7L1

RAB8B

RABGAP1L

RALGDS

RAPGEF3

RASGRF2

RBMS3

RCAN2

RECK

RERG

RFWD2

RGN

RGNEF

RHBG

RHOBTB3

RIEG2

RNASE1

RNASEL

RNF11

ROBO3

RORA

RP9

RPL21

RPS6KA3

RTN4

RUNX1

RWDD1

RYR2

S1PR1

SAMD9L

SBDS

SBDSP

SCARB1

SCD5

SCN3B

SCPEP1

SCRN1

SDCBP

SDPR

SEC31A

SELE

SEMA6D

SEPP1

SERINC3

SERPINA3

SERPINE2

SERPINF1

SERPINI1

SESN1

SETDB2

SETMAR

SGCD

SGCE

SH3BP5

SH3D19

SH3GLB1

SIGLEC1

SIGLEC11

SIGLEC16

SIRPA

SKAP2

SLC16A4

SLC18A1

SLC23A1

SLC24A6

SLC25A12

SLC25A28

SLC38A2

SLC41A1

SLC46A1

SLC4A3

SLCO3A1

SMARCD3

SMC5

SMOC2

SNAP25

SNCA

SNED1

SNRPN

SNX3

SOBP

SOCS5

SP100

SPANXC

SPG20

SPTAN1

SPTBN1

SRGAP1

SRGAP2

ST3GAL4

ST6GALNAC5

ST7L

STAB1

STEAP1

STEAP2

STIM2

SYNC1

SYNPO

SYTL2

TACC1

TAGLN

TAOK3

TBC1D4

TCEAL1

TCF21

TDGF3

TFE3

TGFBR3

THBS4

TIMP1

TIMP4

TK1

TM9SF2

TMEM159

TMEM176A

TMEM176B

TMEM22

TMEM47

TNFSF13B

TNFSF14

TNMD

TNPO1

TNS1

TNXB

TOM1L2

TOR1AIP1

TPK1

TPM2

TPP1

TPSAB1

TRIO

TRPC1

TRPM7

TRPV2

TSC22D3

TSHZ2

TSPAN18

TSPAN2

TSPAN4

TSPAN5

TXNRD1

UBA2

UBA7

UBASH3A

UCHL1

USP21

VAPA

VCAM1

VIPR2

VIT

VWF

WASF3

WASL

WDR26

WFIKKN2

WIPF3

WISP2

WNK1

WNK3

WNT2B

WWTR1

ZBTB43

ZC3H12C

ZEB2

ZFPM2

ZNF521

ZSWIM5
