# Supplementary material for: Endometriosis Gene Expression Heterogeneity and Biosignature: A Phylogenetic Analysis
Source: Obstet Gynecol Int. 2011 Dec 13;2011:719059. doi: 10.1155/2011/719059 (PMC3238413; doi:10.1155/2011/719059)
Supplement: Supplementary file 2 [file 719059.f2.doc]

Supplemental 2: Underexpressed genes among the 1923 synapomorphies of the upper 4 specimens of endometriosis.

7A5

ACACA

ACCN1

ACSL5

ADAMTS9

ADAT2

ADRA2C

AGPAT2

AGR2

AGRN

AHCY

AHCYL2

ALCAM

ALDH18A1

ALDH1A2

AMD1

ANAPC4

ANK3

ANP32A

AP1M2

APBA3

APCDD1

APEX1

ARF5

ARHGAP11A

ARHGAP8

ARSD

ATP6V0A2

ATP6V1C2

ATRX

AURKB

BACE2

BAIAP2L1

BDH1

BIRC5

BOK

BTG3

BUB1B

C10orf57

C10orf58

C10orf63

C1orf186

C6orf173

C7orf11

CA12

CA8

CACNA1D

CADPS

CALML4

CAPRIN1

CCBL1

CCNA2

CCNO

CD24

CD248

CDC25A

CDC25C

CDCA2

CDCA3

CDCA4

CDCA7

CDCA8

CDCP1

CDK2AP1

CEACAM1

CEACAM21

CELSR1

CELSR2

CENPE

CENPF

CENPH

CENPK

CHD7

CHEK1

CHMP4C

CITED4

CLDN3

CLDN4

CLDN7

CLMN

CMTM6

CNDP2

CNKSR1

CNTN3

COBL

COL3A1

COX6C

CPXM1

CRB3

CRYM

CSE1L

CSNK1G2

CSTF3

CTNNA2

CTNNBIP1

CXADR

CYB561

CYP2J2

DACH1

DACT2

DDR1

DEGS2

DEPDC1

DEPDC1B

DIO2

DLX5

DLX6

DNAI2

DNAJC14

DPP6

DSPP

DUSP4

E2F2

E2F5

ECM1

EDAR

EFNB2

EHF

ELF3

ELMO3

ELP3

EMG1

EMID1

EMX2

EMX2OS

EPB41L5

ERBB3

ESR1

EVI1

EXOC4

EYA2

FA2H

FANCA

FARP1

FBLN7

FGFR2

FOLH1

FOXA2

FRAS1

FRAT2

FXYD3

FZR1

GABRP

GALNT4

GATA2

GCNT1

GCNT2

GGT1

GINS4

GNAS

GNLY

GPAA1

GPM6B

GPR160

GPR39

GPR64

GPSM2

GRHL2

GTF2I

GTSE1

H2AFY

H2AFZ

HAPLN4

HEY1

HGD

HINT1

HIP1R

HMG20B

HMGA1

HMGB1

HMGB3

HMGN1

HNF1B

HNRNPA3

HNRNPM

HOMER2

HOOK1

HOOK2

HOXA10

HOXA11

HOXA9

HOXB2

HOXB3

HOXB4

HOXB5

HOXB6

HOXB7

HOXB8

HOXD10

HOXD11

HPN

HPSE2

HSD11B2

ICA1

ID1

IDH2

IHPK2

INADL

IRF6

ITGB8

JARID1B

JAZF1

KEAP1

KIAA0194

KIF12

KIF2A

KIF2C

KIF5B

KIFC1

KLHL13

KRT19

KRT8

LAMA5

LAMC2

LAMC3

LARP1

LEF1

LIMS1

LLGL2

LPAR1

LPAR3

LPHN3

LRBA

LSR

MAD2L2

MAL2

MANSC1

MAP2K6

MAP3K1

MARK1

MARVELD2

MCCC2

MCF2L

MCM10

MCM4

MCM8

MED28

MEIS1

METAP1

METTL9

MFSD2

MGC4172

MKI67

MLLT4

MMP12

MMP26

MMRN2

MOXD1

MPZL2

MREG

MSX1

MUC1

MXD3

MYLIP

MYO5B

NAP1L4

NCAPG2

NCAPH

NCOA6

NDE1

NDUFB11

NELL1

NFYA

NFYB

NLGN1

NPR2

NR2F6

NRTN

NRXN1

NTN1

NUP98

OTUB1

OVOL2

OXR1

P2RY14

PAIP1

PAK6

PAPSS1

PAX2

PAX8

PCBD1

PCIF1

PCOLCE

PDE4A

PDIA3

PDLIM1

PERLD1

PERP

PFKL

PHACTR4

PIAS3

PIK3R1

PKP3

PLCH1

PLK1

PLLP

PLSCR3

PMAIP1

PODXL

POLD1

POLE

POU5F1

POU5F1P1

POU5F1P3

PPAP2A

PPAP2C

PPM1H

PPP2R2C

PRDM1

PRIM2

PRKAB1

PRSS16

PRSS8

PSMD2

PSME4

PTBP1

PTCH1

PTGR1

PTMA

PTPN3

PYY

RAB11FIP4

RAB25

RAB40C

RAD51L3

RAD54B

RAMP1

RASEF

RBBP4

RBBP8

RBM35A

RCAN3

RCC1

REV3L

REXO2

RFWD2

RGL3

RHBDL2

RHOF

RHPN2

RIPK2

RIPK4

RORB

RPLP0

RTKN

RUSC1

RXFP1

RXRB

S1PR3

SALL1

SCARA3

SCGB2A1

SCNN1A

SEMA3F

SEMA5A

SEPT11

SERPINA1

SFPQ

SHANK2

SHROOM3

SIX4

SLBP

SLC11A2

SLC1A2

SLC22A5

SLC24A3

SLC26A2

SLC26A7

SLC2A1

SLC35A2

SLC37A3

SLC37A4

SLC39A6

SLC44A4

SMARCB1

SMARCC1

SMC2

SNRPE

SOCS2

SOX17

SPA17

SPAG1

SPAG5

SPATS2

SPC25

SPDEF

SPINT1

SPINT2

SPTLC2

SRGAP2

SRPK1

SSFA2

SSX2IP

ST14

ST6GALNAC1

ST6GALNAC2

ST8SIA2

STIL

STK25

STRA6

STRBP

STT3A

STXBP6

TACSTD1

TAF7L

TBX2

tcag7.1238

TCF12

TCF7L1

TEAD2

TELO2

TFAP2C

TFCP2L1

TK1

TLE1

TMEFF1

TMEM16D

TMEM30B

TMEM48

TMPRSS2

TNNI3

TP53

TPD52

TPD52L1

TPX2

TRAF4

TRAK1

TRD@

TRIM11

TRIM32

TRIP6

TROAP

TRPM4

TSHZ3

TSPAN1

TSPAN13

TSPAN14

TUBGCP4

TWIST2

TXK

TXN

TYMS

UBE2G1

UCP2

UGT8

VAV2

VCAN

VTCN1

WBP11

WDR63

WEE1

WHSC1

WNT2

XIC

XPR1

YIPF3

ZMYND8

ZNF217
