# Supplementary material for: Endometriosis Gene Expression Heterogeneity and Biosignature: A Phylogenetic Analysis
Source: Obstet Gynecol Int. 2011 Dec 13;2011:719059. doi: 10.1155/2011/719059 (PMC3238413; doi:10.1155/2011/719059)
Supplement: Supplementary file 3 [file 719059.f3.doc]

Supplemental 3: Synapomorphies for the three transitional specimens: GSM 75783, 75784, and 75785.

AAAS

AAMP

ABCB1

ABO

ABRA

ACAD9

ACLY

ACP1

ACTR2

ADAM10

ADAM23

ADAMTS13

AGL

AGXT

AHNAK

AK5

AKAP9

ALKBH5

ALPI

ANGEL2

ANK1

ANKFY1

ANKH

ANKRD40

ANKS1B

ANXA7

AP1G1

API5

APLP2

APOA4

APOBEC4

APOM

ARHGEF12

ARHGEF7

ARHGEF7

ARID5B

ARMC2

ARMCX5

ARX

ASCC3L1

ASXL1

ATAD2B

ATF2

ATOH8

ATP10A

ATP13A4

ATP2C1

ATP8B1

ATXN8OS

AXIN2

BAT2D1

BAT5

BCGF1

BCL10

BCL2

BCL2L14

BCL9L

BEST1

BRAP

BRD4

BSDC1

BTG1

C10orf47

C10orf76

C10orf90

C11orf70

C14orf142

C16orf42

C16orf70

C17orf50

C17orf80

C19orf6

C19orf61

C1orf108

C1orf116

C1orf190

C1orf52

C1orf63

C1orf69

C1orf96

C1QTNF5

C20orf107

C21orf2

C21orf37

C21orf58

C2orf25

C2orf47

C3orf58

C3orf63

C6orf134

C6orf195

C6orf199

C7orf46

C8orf74

C8orf81

C9orf102

C9orf102

C9orf144

C9orf44

C9orf47

C9orf64

CA1

CA13

CACNA1C

CAMK2A

CAMLG

CASR

CASZ1

CATSPER2

CBL

CBWD1

CBX5

CCDC120

CCDC52

CCDC6

CCNT2

CD163L1

CD44

CD46

CD46

CD70

CDC16

CDC40

CDC42BPA

CDC42SE2

CDH19

CDH6

CDK3

CDK5R1

CDK8

CDYL2

CECR2

CENPJ

CERCAM

CFHR4

CHD1L

CHP

CHRNA9

CHST11

CHST9

CLEC4G

CMTM5

CNOT3

COL13A1

COL21A1

COL27A1

COL3A1

COL9A3

COLEC10

CORO2A

COX19

CPD

CPD

CPS1

CPSF1

CRISP3

CSF3R

CSNK1A1

CSNK1A1

CSNK2A1

CSPP1

CTNND1

CTNND1

CTPS2

CYB561

CYCS

DAPK2

DAZ4

DBT

DDA1

DDHD1

DDX52

DGCR12

DHODH

DHX15

DHX38

DHX9

DICER1

DICER1

DKFZP564O0823

DLAT

DNAJB1

DNAJC18

DNAJC6

DNAL1

DPEP2

DPY19L1

DR1

DST

DYNC1LI1

EFHB

EFHD1

EHD2

EHD2

EHHADH

EIF1

EIF4E2

EIF4G2

ELAVL3

ELMOD2

ENOSF1

EPB41L4B

EPOR

ERAP1

ESR2

EVI2A

EXOC2

EXOC4

F2RL2

F2RL2

FAHD2A

FAM149B1

FAM161A

FAM163A

FAM69A

FER1L3

FEZF2

FGF13

FGF21

FKBP15

FLJ20518

FNBP4

FOSL2

FOXA2

FOXC2

FOXR2

FUNDC2

GABRB2

GADD45B

GAGE4

GAN

GATA6

GATAD1

GBA2

GCG

GFPT1

GGA2

GGT1

GJD4

GLG1

GLS

GLTP

GNAI2

GNAS

GNPDA1

GOLGA2L1

GPR133

GPR26

GPR63

GRASP

GRIN1

GRIN3B

GRK5

GTF2I

GUF1

GYG2

H1F0

HGF

HHAT

HIBADH

HIF3A

HINT1

HIP1

HIRA

HIST1H2BH

HIVEP1

HLA-J

HLCS

HMGA2

HNRNPD

HNRNPM

HOXB13

HPCAL4

HSPA12A

HTR5A

ICMT

IFT57

IGSF22

IL1RAP

IL1RN

IL21R

IL2RA

INADL

INHBA

INTS10

IRG1

JARID1A

JHDM1D

JMJD6

KBTBD4

KCMF1

KCNAB1

KCNAB2

KCNH5

KEL

KIAA0415

KIAA0494

KIAA0746

KIAA1009

KIAA1128

KIAA1217

KIAA1217

KIAA1804

KIAA1967

KIFC2

KLHDC1

KLHL9

KRT13

KRT40

KRT5

KRTAP4-3

KRTCAP2

L3MBTL

LACTB

LAMA2

LAMC2

LAMP1

LANCL2

LARGE

LARP5

LARS2

LDLR

LIFR

LILRB2

LIPT1

LIX1L

LOC100129289

LOC150383

LOC200383

LOC200383

LOC202459

LOC388946

LOC440354

LOC642558/FUSIP1

LOC646756/FOXRED2

LOC646934

LOC728853

LONP2

LRPPRC

LRRC8C

LRRN1

LRRN2

MAF1

MAFG

MAGOH2

MAN1A2

MANEA

MAP2K5

MAPK1IP1L

MARK3

MARS

MAZ

MDFIC

MDGA1

MDS2

MEGF9

MEOX1

METT11D1

MGA

MGC34774

MIA3

MIDN

MINK1

MINK1

MIST

MKNK1

MMP28

MOBKL1B

MOCS3

MORC1

MPHOSPH10

MPP1

MPP6

MRO

MRPL1

MRPL10

MRPL43

MRPL44

MTF1

MTHFD2L

MTMR4

MUC16

MYBPH

MYCT1

MYH7

MYLIP

MYO16

MYO1G

MYO6

MYO7A

MYOG

MYSM1

MYSM1

N4BP2

N6AMT1

NADSYN1

NARG1

NARG1L

NAT8B

NAV1

NBR2

NCAM1

NCKAP1L

NCOA7

NENF

NFX1

NGFR

NIPBL

NKAIN4

NPAS4

NPCDR1

NPFF

NSL1

NSUN5B

NUDT4P2/NUDT4

NUMB

OR5K1

ORC5L

OSGIN1

OSGIN2

OTUB2

OXCT2

P2RX1

PAFAH1B1

PAIP1

PALMD

PAPOLA

PARN

PBLD

PBXIP1

PCBP2

PCDHB10

PCDHB19P

PCDHGC3

PCDHGC5

PCID2

PCMT1

PCSK6

PDE4D

PDIA3

PDS5A

PDXDC1

PEX1

PGPEP1

PHF12

PHF13/THAP3

PIAS2

PIK3C2A

PIK3IP1/RNF185

PKN1

PLEKHA5

PMS2L3

PMS2L3

PNO1

PODXL2

POLE4

POLE4

POLR1B

POLR2K

POLR3H

PPIC

PPID

PPM1A

PPP3CC

PPP3R2

PRM1

PRMT2

PROSC

PRPF18

PRPS2

PRRC1

PSCD4

PSEN2

PTPRC

PXMP3

QSER1

RAB1A

RAB3IP

RABL3

RANBP10

RANBP17

RANBP3

RARG

RASL11B

RBL1

RBM25

RBM41

RBMS3

RBPMS

RCL1

RDH10

RECQL5

REM2

REXO1

RFNG

RFX3

RGS3

RGS5

RHOB

RICH2

RIMS1

RIOK2

RLTPR

RNF141

RNF180

RNF180

RNF44

RNFT1

RP1-21O18.1

RPGRIP1L

RPL35

RPP30

RPS15A

RPS19

RTKN2

RUNX1

RUNX1T1

S1PR4

SBK1

SCARB2

SCML4

SCYL2

SEC61A2

SEL1L2

SEPSECS

SEPT2

SEPT2

SERAC1

SERHL2

SERPINF2

SERTAD2

SETD2

SF3B3

SFRS12

SGCB

SGEF

SGPP1

SGPP1

SGSM3

SH2D4B

SHANK2

SLAIN2

SLC18A1

SLC1A4

SLC1A5

SLC22A15

SLC25A37

SLC25A45

SLC27A2

SLC2A2

SLC30A1

SLC31A2

SLC33A1

SLC37A1

SLC44A2

SLC45A2

SLC45A3/PCANAP8

SLFN5

SMEK2

SMNDC1

SMPDL3B

SNHG4

SNRPA1

SNX21

SOCS3

SOCS5

SOHLH2

SORT1

SPAG9

SPAST

SPATA9

SPEG

SPOCK3

SPRY2

SQSTM1

SQSTM1

SRCRB4D

SRGAP1

SRP72

SSX3

ST3GAL3

ST8SIA3

STAM2

STK36

STK36

STOX2

STX16

STYX

SUB1

SUMO2

SUPT3H

SUZ12P

SVEP1

SYAP1

SYDE1

SYNE1

SYNGR3

SYTL3

SYTL3

TAL2

TARS2

TAS2R45

TBC1D8B

TBKBP1

TBRG1

TBX10

TC2N

TCF12

TCF7L1

TCL6

TCOF1

TEGT

TFAP2E

TFG

TGFB1I1

THAP5

THPO

THSD7A

THUMPD3

TIFA

TIRAP

TLX3

TM7SF3

TMCO4

TMCO5A

TMED2

TMEM102

TMEM106A

TMEM143

TMEM157

TMEM164

TMEM168

TMEM170A

TMEM178

TMEM41B

TMEM44

TMOD2

TNFRSF10D

TNFRSF11A

TNIP3

TPRG1L

TRIM5

TSC22D2

TSC22D3

TSPAN15

TSPAN16

TSPYL5

TTC12

TWF1

UBE2J1

UBE2L6

UBE2U

UBE3A

UGCGL2

UNC45A

UNC50

UQCC

UQCC

USF2

USP33

UTRN

VAMP7

VAV3

VAV3

VCY

VENTX

VSX1

VTI1A

WASF2

WASL

WDR33

WDR8

WFIKKN2

WIF1

WIPF1

WNT7A

WNT7B

XIAP

XIST

YES1

YTHDC2

YTHDC2

ZC3HAV1

ZCCHC10

ZCRB1

ZDHHC1

ZDHHC13

ZDHHC20

ZDHHC20

ZDHHC21

ZDHHC22

ZFP1

ZFP28

ZNF195

ZNF358

ZNF395

ZNF395

ZNF41

ZNF445

ZNF587

ZNF599

ZNF682/ZNF90

ZNF688

ZNF765

ZNF829

ZNRF2

ZSCAN20
